# High-Branched Organosilicon Epoxy Resin with Low Viscosity, Excellent Toughness, Hydrophobicity, and Dielectric Property

**DOI:** 10.3390/molecules28062826

**Published:** 2023-03-21

**Authors:** Min Yu, Zeyuan Chen, Jie Li, Jihuai Tan, Xinbao Zhu

**Affiliations:** 1 Jiangsu Co-Innovation Center of Efficient Processing and Utilization of Forest Resources, College of Chemical Engineering, Nanjing Forestry University, Nanjing 210037, China; 2 Anhui Engineering Research Center of Epoxy Resin and Additives, Huangshan 245900, China

**Keywords:** silicone resins, viscosity reduction, toughening, hydrophobicity, dielectric properties

## Abstract

Rapidly developing technology places higher demands on materials, thus the simultaneous improvement of materials’ multiple properties is a hot research topic. In this work, a high-branched silicone epoxy resin (QSiE) was synthesized and applied to the curing system of bisphenol A epoxy resin (DGEBA) for modification investigations. When 6 wt% QSiE was added to the system, the viscosity dropped by 51.8%. The mechanical property testing results indicated that QSiE could significantly enhance the material’s toughness while preserving good rigidity. The impact strength was enhanced by 1.31 times when 6wt% of QSiE was introduced. Additionally, the silicon skeleton in QSiE has low surface energy and low polarizability, which could endow the material with good hydrophobic and dielectric properties. This work provided a new idea for the preparation of high-performance epoxy resin additives, and provided a broad prospect for cutting-edge applications of epoxy resins.

## 1. Introduction

Epoxy resins, one of the most important kinds of thermosetting resins [1,2], are extensively applied in coatings, adhesives, composites and electronic packaging due to their excellent mechanical properties, chemical resistance, adhesion capabilities and processability [3,4]. During curing, epoxy resins cross-link to produce a tight three-dimensional molecular network structure, which provides the material with high stiffness but also leads to substantial brittleness, severely limiting its further applications. As a consequence, a substantial amount of research has been devoted to enhancing the toughness of epoxy resins and composites [5,6].

The most popular and straightforward method to enhance the toughness of epoxy resins and their composites is to add toughening agents. Toughening agents such as nano-inorganics, rubbers, thermoplastic resins and core-shell particles have been reported to enhance the toughness of epoxy resins by forming phase separation in its curing network [7,8,9]. Unfortunately, although these toughening agents improve the toughness to a certain extent, other properties, such as glass transition temperature (T_g_), stiffness and processability, are affected [10]. Additionally, with the advancement of technology, it has become more difficult to fulfill the demands for material production with a single aspect of performance improvement. Epoxy resins need to have enhanced qualities such as hydrophobicity and dielectric properties, in addition to the regular toughening [11,12,13,14]. Therefore, additives that can simultaneously enhance the characteristics of epoxy resins have a substantial business potential and research value.

Due to their unique structure and properties, silicone resins are a highly sought-after toughening agent for epoxy resins. When exposed to external stresses, the soft Si-O bonds in its structure provide good elasticity of expansion and contraction and dissipate external forces via deformation. In addition, the distinctive qualities of silicone resins, such as outstanding resistance to high and low temperatures, electrical insulation, ageing resistance and low surface tension, distinguish them from the several sources used to produce multifunctional epoxy resins [15,16,17,18]. Liu et al. [19] prepared hyperbranched polysiloxanes containing Si-O-C (HSiEP) and copolymerized them with bisphenol A isocyanates. The peak curing temperature was decreased by 93.5 °C when 6 wt% HSiEP was incorporated, which considerably enhanced processability. Compared to the pure sample, the flexural and impact strengths were raised by 49.2% and 105.2%, respectively. Moreover, the low polarized C-Si bond and cavity shape of HSiEP imparted the system with excellent dielectric properties, making it potentially promising for applications in electronic packaging. Shi et al. [20] synthesized epoxy modifiers by grafting compounds prepared from aminopropyltrimethoxysilane and valeryl chloride onto silicone polymers. The cured thermosets significantly enhanced their tensile and impact properties while remaining heat resistant. When added at 3 wt%, the impact strength of cured thermosets was increased by 18.2%. Zheng et al. [21] developed a series of MQ silicone resins for the toughening of epoxy resins, and the results demonstrated that the incorporation of silicone resins significantly improved the toughness, thermal stability, and water resistance of the cured composites. The addition of 20 wt% MQ resin enhanced impact strength by 90%, while the composites showed improved hydrophobicity (contact angle increased from 80° to 110°) and thermal stability due to the silicone fraction’s low polarity and high thermal stability. The research mentioned above demonstrated that silicone resins, because of their inherently flexible structural and unique properties such as low surface tension and low polarization, can enhance the multifaceted performance of materials while imparting good toughness.

In this work, hydrogen-containing silanes with high degree of branching (QSiH) were prepared by the equilibrium reaction of tetramethoxysilane with terminal hydrogen-containing silanes. Highly branched silicone epoxy resins (QSiE) were then produced by the introduction of epoxy groups via the silicon-hydrogen addition reaction of the Si-H bond in QSiH to the double bond in allyl glycidyl ether. To carry out the modification investigation, QSiE was blended in various ratios with the bisphenol A epoxy resin/4,4-diaminodiphenylmethane (DGEBA/DDM) curing system. The influence of QSiE on the processability of the material was examined by measuring the system’s viscosity at various doping levels. In order to determine how QSiE affects the overall properties of the epoxy resin, the mechanical properties, thermal stability, hydrophobicity and dielectric properties of the cured products were also investigated. The purpose of this work is to widen the application areas of epoxy resins by improving several properties and reducing the inherent brittleness drawbacks, as well as to propose a novel idea for the design of high-performance and multifunctional epoxy resin additives.

## 2. Results

### 2.1. Structural Characterizations

**Figure 1:**
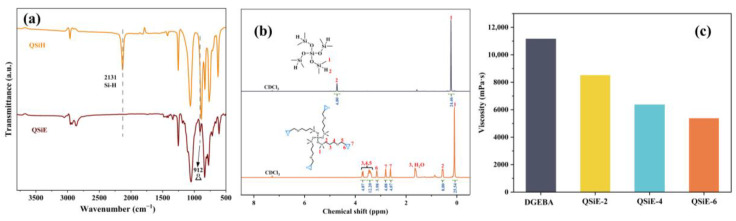
The FTIR spectra (**a**) and ^1^H NMR spectra (**b**) of QSiH and QSiE, (**c**) Viscosity-reduction effect of QSiE.

The structures of QSiH and QSiE were verified by FTIR and ^1^H NMR. The FTIR spectra of QSiH and QSiE was shown in Figure 1a. In contrast to QSiH, the characteristic peak of Si-H at 2131 cm^−1^ disappeared in the QSiE spectrum, whereas the characteristic peak of the epoxy group was observed at 912 cm^−1^ [22]. This indicated that the hydrosilylation reaction was carried out successfully. Additionally, ^1^H NMR was used to corroborate the structures of QSiH and QSiE, and the results were shown in Figure 1b. In the QSiE spectrum, the characteristic peak of Si-H (4.7 ppm) disappeared completely, while new absorption peaks appeared between 2.6 and 3.1 ppm, which belonged to the epoxy group [23]. Furthermore, the chemical shifts and integrated areas of all signals in the QSiE spectrum conform to their theoretical values. The aforementioned results indicated that both QSiH and QSiE have been successfully synthesized and exhibit accurate structures.

### 2.2. Viscosity-Reducing Ability

An appropriate viscosity is advantageous for the application of epoxy resins in industrial production as it is one of the most significant indications of the performance of epoxy resins [24]. However, DGEBA has a high viscosity of its own, reaching 11,160 mPa-s at room temperature (25 °C), making it challenging to process. As a result, diluents must frequently be added while using DGEBA in order to decrease viscosity and enhance flow and processability. Although these additives enhance the processability to a certain extent, other properties, such as thermal stability, mechanical properties and glass transition temperature, are decreased. QSiE has a viscosity of only 128 mPa·s at room temperature (25 °C) due to its highly branched and multiple epoxy groups. Therefore, QSiE can be utilized to toughen and reduce viscosity at the same time. Figure 1c depicts the viscosity of the system after adding various amounts of QSiE. The viscosity of the system was greatly reduced with the addition of QSiE, and when the incorporation amount reaches 6 wt%, the viscosity can be reduced by 51.8%, indicating a strong viscosity reduction impact. By lowering the system’s viscosity, QSiE can effectively enhance the processability of epoxy resins, decreasing the requirement for additional diluents in the system and enhancing convenience of use.

### 2.3. Thermal Stability

**Figure 2:**
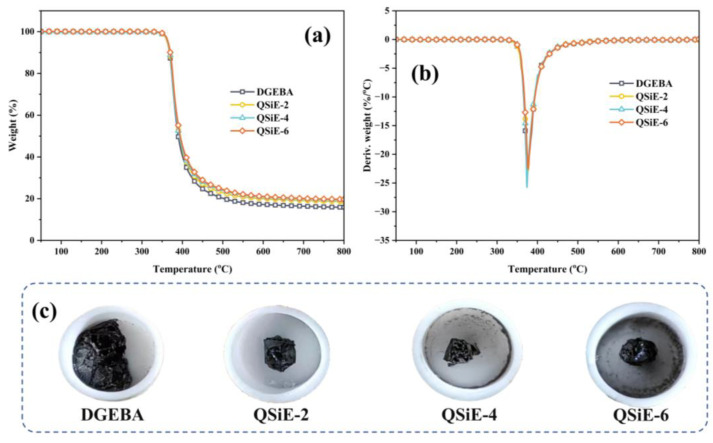
TG (**a**) and DTG (**b**) curves of cured thermosets, photos of samples (**c**) after TGA tests.

Currently, the majority of toughener and diluent capabilities are frequently obtained at the expense of the material’s thermal stability. The thermal stability of the cured thermosets under a N_2_ atmosphere was investigated by thermogravimetry. The TG and DTG curves were shown in Figure 2a,b, and the specific parameters were listed in Table 1. As shown in Figure 2a, the QSiE/DGEBA system has only one degradation phase under a nitrogen atmosphere, similar to that of pure DGEBA. The incorporation of QSiE had no effect on the initial decomposition temperature (T_5%_) of cure thermosets, and shows good thermal stability. The thermal stability of cured thermosets following QSiE incorporation increases slightly as the temperature rises. Compared with pure DGEBA, the residual carbon rate at 800 °C increased from 15.8% to 18.2% at a doping level of only 2 wt%. This is because the silicon skeleton of the QSiE structure produced an inorganic SiO_2_ structure that can stabilize at high temperature, thus improving the residual carbon rate at 800 °C [25]. The sample’s morphology after the TG test was shown in Figure 2c, where the DGEBA carbon layer has a flaky morphology and broke up when lightly touched. On the contrary, the QSiE-doped samples exhibited a dense spherical carbon layer that separated the outside from combustion and oxygen migration to the interior, preventing further matrix degradation and enhancing the material’s thermal stability [26,27].

### 2.4. Mechanical Properties

**Figure 3:**
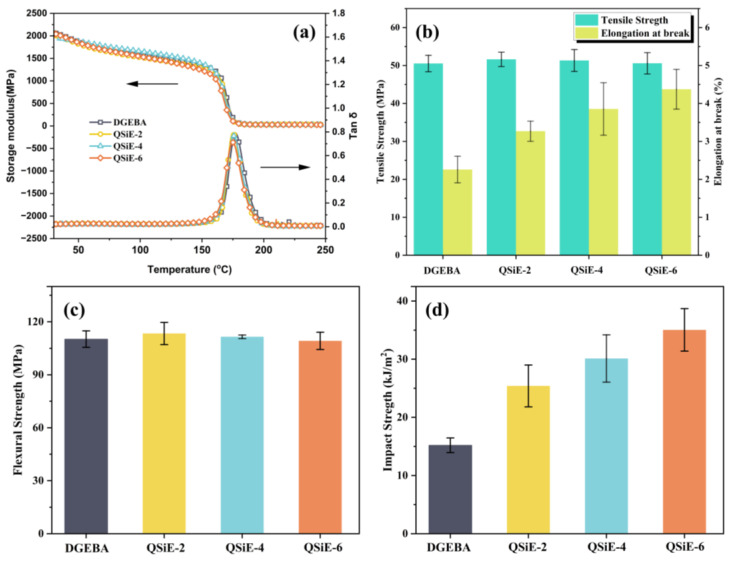
Storage modulus (**a**), Tan δ (**b**), tensile strength and elongation at break (**c**), and impact strength (**d**) of cured thermosets.

The effect of QSiE incorporation on the thermomechanical properties of the cured thermosets was investigated by DMA and the results were shown in Figure 3a,b, and the specific data were listed in Table 2. After the addition of QSiE, the storage modulus of the cured thermosets at room temperature was substantially equivalent to that of DGEBA, maintaining good stiffness. As noted in Figure 3b, there was just one peak in Tan δ for all cured thermosets, indicating that QSiE was able to participate effectively in the curing system and that there was no phase separation [28]. Furthermore, when compared to DGEBA, the glass transition temperature (T_g_) of the modified cured products showed essentially no decrease, and even with the addition of 6 wt%, the T_g_ only decreased by 2.6 °C, indicating excellent heat resistance. Generally, the use of toughening agents decreases the stiffness of system, particularly when soft silicone resins are added, which will reduce the T_g_ of cured thermosets. To better explain this phenomenon, the crosslink density *ν_e_* of the cured thermosets was calculated by following equation [29].

*ν_e_* = *E’*/3RT (1)

where *E’* is the storage modulus at T_g_ + 30 °C, R is the universal gas constant and T is the absolute temperature (T_g_ + 30 °C). The calculated *ν_e_* of the cured thermosets was listed in Table 2. The *ν_e_* for DGEBA is 2.32 × 10^3^ mol/m^3^, while the *ν_e_* of QSiE-2, QSiE-4 and QSiE-6 was 2.56, 2.29 and 2.34 × 10^3^ mol/m^3^, respectively. It can be shown that the crosslink density of the system does not decrease with the addition of QSiE, but rather slightly rises. As crosslinking density increased, the free volume of the cured product was reduced, but the average chain length of adjacent crosslinking points decreased, resulting in a decrease in the mobility of the chain segments in the cured network. The increase in crosslink density effectively compensated for the loss of stiffness produced by the introduction of flexible Si-O bonds into the system, allowing the cured thermosets to retain a high degree of stiffness after the incorporation of QSiE.

The effect of QSiE on the mechanical properties of the cured thermosets was further investigated by the tensile, flexural and impact tests. The tensile strength and elongation at break of the cured thermosets were shown in Figure 3b; the tensile strength of DGEBA reached 50.5 MPa, indicating good rigidity. The tensile strength of cured thermosets did not significantly decrease with the incorporation of QSiE, which is consistent with the DMA results. Because of the three-dimensional cross-linked network that forms after curing, the application of epoxy resins was limited by brittleness. Therefore, enhancing their toughness is crucial for expanding their application range. When QSiE was added to the system, the elongation at break of cured thermosets significantly increased. For example, the elongation at break of DGEBA was only 2.26%; however, QSiE-6 reached 4.37% with a 93.4% improvement.

The flexural strength of the cured products was depicted in Figure 3c. Flexural strength, like tensile strength, is frequently used to measure the stiffness of epoxy resins. Compared to the pure DGEBA, with the incorporation of 2wt% QSiE, the flexural strength of the cured thermoset was improved from 110.2 MPa to 113.4 MPa. The flexural strength of the cured thermosets was trending downward as the QSiE addition amount increased further. However, even with the addition of 6 wt% QSiE, the flexural strength of the cured thermosets still exhibited good stiffness and was only slightly decreased compared to DGEBA. The flexural strength is mainly influenced by the rigid groups and the crosslinking density. The soft Si-O bonding of QSiE, as well as the free volume due to the highly branched structure, both caused a reduction in the rigidity of the system, but the higher crosslinking density of QSiE incorporation can compensate for the loss of rigidity. As a result, the addition of QSiE within a particular range has no effect on the system’s stiffness.

An impact test was used to determine the toughness of the cured thermosets, and the results are displayed in Figure 3d. The impact strength of DGEBA was just 15.2 kJ/m^2^, indicating significant brittleness. The toughness of cured thermosets increased significantly with the incorporation of QSiE, and the impact strength increased with the QSiE incorporation. The impact strength of QSiE-6 was 35.04 kJ/m^2^, which was 1.31 times higher than that of DGEBA, indicating a significant toughening effect. The results of DMA, tensile, flexural and impact tests indicated that the addition of small amounts of QSiE can provide a significant improvement in toughness without sacrificing the good stiffness of the DGBEA/DDM system.

**Figure 4:**
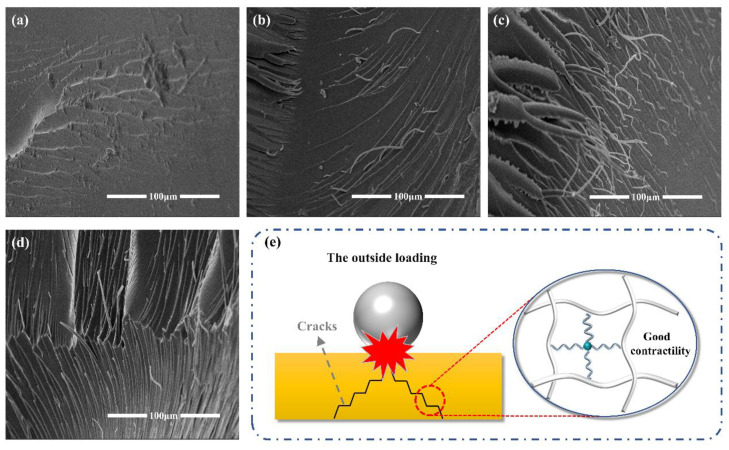
The SEM images of impact fracture surface of (**a**) DGEBA, (**b**) QSiE-2, (**c**) QSiE-4, (**d**) QSiE-6. (**e**) A possible toughening mechanism of QSiE.

To comprehend the toughening mechanism of QSiE, the impact fracture surface of the cured thermosets was analyzed by scanning electron microscopy, and the results are shown in Figure 4. Figure 4a shows that the impact fracture surface of DGEBA was relatively smooth with only a few cracks, indicating a typical brittle fracture. Figure 4b–d depict the impact fracture surfaces of the samples after incorporation of 2 wt%, 4 wt% and 6 wt% QSiE. With the incorporation of QSiE, the fracture surface of the cured thermosets revealed a large number of filaments and cracks, as well as a rougher surface. The excellent toughness effect of QSiE can be explained by the fact that QSiE participated well in the curing of epoxy resins and is distributed uniformly throughout the material. Besides this, in comparison to C-C bonds, the Si-O-Si bonds in QSiE have softer helical structures with longer bond lengths (1.64 A:1.53 A) and bigger bond angles (143°:110°) [30]. Si-O-Si has good elasticity for expansion and contraction under impact or stretching, absorbing external pressures through deformation and enhancing the material’s toughness [31].

### 2.5. Hydrophobic Properties

**Figure 5:**
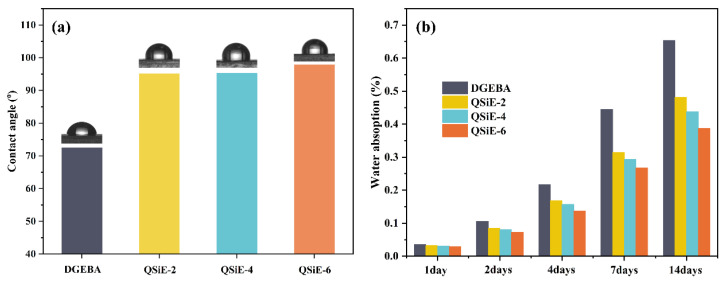
(**a**) Contact angle, (**b**) water absorption rate of cured thermosets.

After the epoxy resins absorbs water, the water molecules cause internal stress and produce a plasticizing effect within the system, leading to a reduction in the mechanical properties and T_g_ of the cured material (20 °C per 1% absorption), limiting the material’s application [32]. Therefore, it is critical to improve the water resistance of epoxy resins in order to broaden the application range of epoxy resins, particularly in fields such as electronics and aerospace. The water absorption of epoxy resins was generally divided into two stages, surface adsorption and internal diffusion, which can be assessed using the water contact angle and the water absorption rate of samples at various immersion times, respectively [33,34]. The contact angle test results of the cured thermosets are shown in Figure 5a. With the addition of QSiE, the hydrophobicity of cured thermosets improved dramatically. With only 2 wt% QSiE incorporation, the contact angle of QSiE-2 could reach 95.2°, demonstrating excellent hydrophobic properties. The water absorption of the cured samples was then further investigated by immersing them in water and measuring the change in mass at various times; the curve of water absorption versus time is shown in Figure 5b. The water absorption of the samples decreased significantly with the incorporation of QSiE. This is due to the low surface tension of the silicone backbone in the QSiE structure, which contributes to its hydrophobic properties. After 14 days of immersion, compared to pure DGEBA, the water absorption of the samples doped with 6 wt% QSiE decreased by 40.8%, which exhibited improved hydrophobicity [35]. The water molecules in the cured thermosets exist in two forms: bound water, which forms hydrogen bonds with water molecules through polar groups in the structure, and free water, which exists in the free volume of the cured thermosets. The QSiE structure contains a low surface tension silicone backbone, which reduces the adhesion of water molecules to the material’s surface. In addition, the increase in cross-link density brought about by the incorporation of QSiE reduced the free volume, hence reducing the amount of free water and enhancing the hydrophobic properties of the materials. These results suggest that QSiE can simultaneously function in both the surface adsorption and internal diffusion phases, thus improving the water resistance of the epoxy resins.

### 2.6. Dielectric Properties

**Figure 6:**
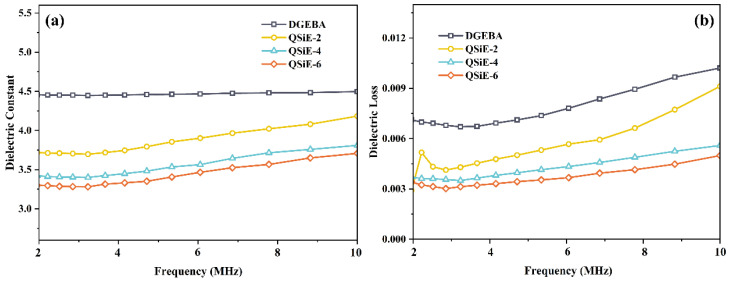
(**a**) Dielectric constant, (**b**) dielectric loss of cured thermosets.

Dielectric properties are one of the important indications of epoxy resins because of their vital function in the electrical and electronic fields. Among the dielectric properties, the dielectric constant and dielectric loss are investigated as two key indicators. The dielectric constant represents the charge storage capacity of the material on the surface, whereas the dielectric loss describes the expenditure of electrical energy in the form of heat by the dielectric in an electric field. Therefore, low dielectric constants and dielectric losses reduce signal propagation delay time and propagation losses, which are critical in lowering equipment heat generation and enhancing data transmission rates [36]. The dielectric constants and dielectric losses of the cured thermosets versus frequency are shown in Figure 6a,b. The dielectric properties of the cured thermosets were greatly enhanced by the incorporation of QSiE. Compared to pure DGEBA, QSiE-6 exhibited a 17.5% reduction in the dielectric constant and a 51.1% reduction in dielectric loss at 10 MHz. The enhanced dielectric properties can be explained by the following factors: the presence of QSiE in the cure reaction introduced a substantial amount of C-Si into the three-dimensional curing network, hence reducing the polarization rate of the resin [37]. Moreover, the highly branched structure of QSiE provided more molecular cavities to the curing system, resulting in a local increase in free volume and the entrance of air, which has the lowest dielectric constant [38]. QSiE is potentially intriguing for applications in the realm of electronics because to its excellent dielectric properties.

## 3. Materials and Methods

### 3.1. Materials

Methyl orthosilicate, tetramethyldisiloxane and allyl glycidyl ether were purchased from Shanghai Maclean Co. Ltd. Sulfuric acid and methanol were purchased from Nanhua Reagent Co. Ltd. Bisphenol A epoxy resin (epoxy value = 0.52 eq/100 g), 4,4-diaminodiphenylmethane (DDM) were supplied by Anhui Xinyuan Technology Co. Ltd. Karstedt catalyst (Pt content: 5000 ppm) was provided by Xilike High-tech Materials Technology Co. Ltd.

### 3.2. Synthesis of QSiE

**Figure 7:**
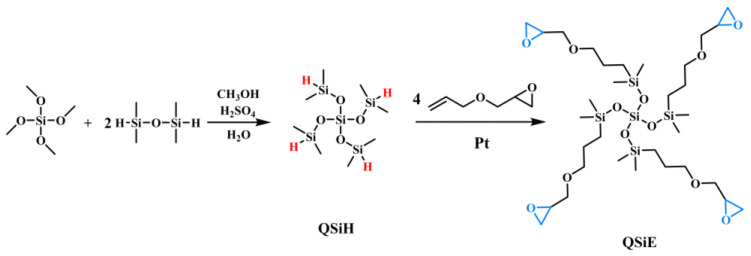
Synthetic route of QSiE.

QSiE was synthesized through a two-step reaction, and the synthetic process is depicted in Figure 7. Firstly, hydrogen-containing silanes with a high degree of branching (QSiH) were prepared by the equilibrium reaction of tetramethoxysilane with terminal hydrogen-containing silanes. Typically, tetramethyldisiloxane (1.5 mol) and sulfuric acid (0.05 mol) was added to a three-necked flask, and methyl orthosilicate (0.5 mol) was added dropwise at 0–10 °C, stirring for 1 h. After that, distilled water (4 mol) was added dropwise to the system and reacted overnight at room temperature. Separating the water layer in the separator funnel, and sulfuric acid (0.05 mol) was added again, followed by stirring for 3 h, separating the sulfuric acid layer, and then distilling at reduced pressure to obtain the low-viscosity liquid QSiH. The purity is 95%, and the yield is 67%.

Following this, QSiE was prepared by a hydrosilylation reaction between QSiH and allyl glycidyl ether. Typically, allyl glycidyl ether (1.1 mol) and Karstedt catalyst (0.2 g) were added to a three-necked flask. QSiE (0.25 mol) was added dropwise to the system at 90 °C, and it was held for three hours. QSiE was obtained by distillation under reduced pressure to remove the excess of allyl glycidyl ether. The FTIR and 1HNMR spectra of QSiH and QSiE are shown in Figure 1.

### 3.3. Preparation of Cured Thermosets

The cured compounds with 2, 4, and 6 wt% QSiE and pure DGEBA were prepared and named QSiE-2, QSiE-4, QSiE-6 and DGEBA, respectively, and the specific compositions are presented in Table 3. QSiE and DGEBA were mixed with theoretical DDM, de-bubbled under a vacuum, poured into a mold, and cured at 100 °C/2 h, 130 °C /2 h, and 160 °C /2 h, in that order. 

### 3.4. Characterization

Fourier transform infrared (FT-IR) spectroscopy was obtained by Bruker spectrophotometer (VERTEX 80 V) from 4000 to 500 cm^−1^ wavenumbers, and scanning time was 32 at a resolution of 4 cm^−1^. ^1^HNMR spectroscopy was performed by a 600 MHz superconducting NMR spectrometer (Bruker AVANCE III HD), with deuterated DMSO as a solvent. The viscosity of the system was obtained by testing with a viscometer (Brookfield DVPlus) at room temperature (25 °C). Thermogravimetric analysis (TGA) was conducted by Shimadzu thermal analyzer (DTG-60AH) under an N_2_ atmosphere (50 mL/min) and heated from 30 to 800 °C with a heating rate of 10 °C/min. Dynamic mechanical analysis (DMA) was carried out by TA Q800, from 30 to 220 °C with a heating rate of 3 °C/min, using specimens of 60 × 10 × 4 mm^3^ in size. Tensile properties were examined using a Universal Testing Machine (E432.104) according to the GB/T 1040.1–2006 with a testing rate of 10 mm/min, and the tensile strength and elongation at break were obtained by calculating the average of 5 samples. The testing of flexural properties was conducted by ETM504C microcomputer-controlled electronic universal testing machine according GB/T2567-2008, with a sample dimension of 80 × 15 × 4 mm^3^, and the flexural strength was obtained by calculating the average of 5 samples. XJJ-5 Impact Testing Machine was used to evaluate impact strength referring to the GB/T 2567–2008 with a sample dimension of 80 × 10 × 4 mm^3^ without notch, and impact strength was obtained by calculating the average of 5 samples. The analysis of the fracture surface morphology of samples after impact testing was performed on a Hitachi S-4800 scanning electron microscope (SEM) operated at 1 kV. The contact angle was carried out on a KRUSS DSA100 at room temperature, and the volume of water was set at 10 μL. The water absorption ratio was obtained by submerging the sample entirely in water and weighing the change in mass of the sample at different times. The dielectric properties were measured by a dielectric spectrometer (Agilent 4294A) in the frequency range of 2- 10 MHz at room temperature (25 ^o^C) with a sample dimension of 10 × 10 × 2 mm^3^.

## 4. Conclusions

In this work, a high-branched silicone epoxy resin was synthesized to simultaneously improve the multiple properties of epoxy resins. Thanks to the lower viscosity of QSiE, the viscosity of the system decreased by 51.8% at a doping level of 6 wt%. The QSiE structure contains a stable Si-O bond, which enhanced the residual carbon rate at high temperatures. The DMA and mechanical properties results demonstrated that QSiE could significantly enhance the material’s toughness and preserve good rigidity. Due to the soft Si-O structure and the efficient dispersion in the DGEBA/DDM curing system, the impact strength was improved by 1.31 times when 6 wt% QSiE was incorporated. Additionally, the silicon skeleton in QSiE also has low surface energy and low polarizability, which could endow the material with good hydrophobic and dielectric properties. This work offers ideas for the preparation of high-performance epoxy resins, which hold great potential in frontier fields.

## Figures and Tables

**Table 1:** Thermogravimetric data of cured thermosets.

Sample	T_10%_ (°C)	T_max_ (°C)	C_y800_ (%)
DGEBA	368.1	374.4	15.9
QSiE-2	368.7	376.0	18.7
QSiE-4	369.1	374.0	19.5
QSiE-6	369.9	376.8	19.7

**Table 2:** Thermal-mechanical properties of cured thermosets.

Sample	T_g_ (°C)	Storage Modulus at 35 °C (MPa)	*ν_e_* (×10^3^ mol/m^3^)
DGEBA	177.4	2033.7	2.32
QSiE-2	175.1	1978.9	2.56
QSiE-4	175.9	1943.1	2.29
QSiE-6	174.9	2012.1	2.34

**Table 3:** The specific formulas of cured thermosets.

Sample	QSiE Content (%)	DGEBA (g)	QSiE (g)	DDM (g)
DGEBA	0	100	/	25.77
QSiE-2	2	100	2	26.28
QSiE-4	4	100	4	26.78
QSiE-6	6	100	6	27.29

## Data Availability

All data are included in the manuscript.
